# In vitro assessment of antibody-conjugated gold nanorods for systemic injections

**DOI:** 10.1186/s12951-014-0055-3

**Published:** 2014-12-05

**Authors:** Sonia Centi, Francesca Tatini, Fulvio Ratto, Alessio Gnerucci, Raffaella Mercatelli, Giovanni Romano, Ida Landini, Stefania Nobili, Andrea Ravalli, Giovanna Marrazza, Enrico Mini, Franco Fusi, Roberto Pini

**Affiliations:** Dipartimento di Scienze Biomediche Sperimentali e Cliniche ‘Mario Serio’, Università degli Studi di Firenze, Viale Pieraccini 6, 50139 Firenze, Italy; Istituto di Fisica Applicata ‘Nello Carrara’, Consiglio Nazionale delle Ricerche, Via Madonna del Piano 10, 50019 Sesto Fiorentino, Italy; Dipartimento di Chimica ‘Ugo Shiff’, Università degli Studi di Firenze, Via della Lastruccia 3, 50019 Sesto Fiorentino, Italy; Dipartimento di Scienze della Salute, Università degli Studi di Firenze, Viale Pieraccini 6, 50139 Firenze, Italy; Dipartimento di Medicina Sperimentale e Clinica, Università degli Studi di Firenze, Largo Brambilla 3, 50134 Firenze, Italy

**Keywords:** Gold nanorods, Cancer antigen 125, Active targeting, Competitive assay, Matrix effect, Blood compatibility

## Abstract

**Background:**

The interest for gold nanorods in biomedical optics is driven by their intense absorbance of near infrared light, their biocompatibility and their potential to reach tumors after systemic administration. Examples of applications include the photoacoustic imaging and the photothermal ablation of cancer. In spite of great current efforts, the selective delivery of gold nanorods to tumors through the bloodstream remains a formidable challenge. Their bio-conjugation with targeting units, and in particular with antibodies, is perceived as a hopeful solution, but the complexity of living organisms complicates the identification of possible obstacles along the way to tumors.

**Results:**

Here, we present a new model of gold nanorods conjugated with anti-cancer antigen 125 (CA125) antibodies, which exhibit high specificity for ovarian cancer cells. We implement a battery of tests *in vitro*, in order to simulate major nuisances and predict the feasibility of these particles for intravenous injections. We show that parameters like the competition of free CA125 in the bloodstream, which could saturate the probe before arriving at the tumors, the matrix effect and the interference with erythrocytes and phagocytes are uncritical.

**Conclusions:**

Although some deterioration is detectable, anti-CA125-conjugated gold nanorods retain their functional features after interaction with blood tissue and so represent a powerful candidate to hit ovarian cancer cells.

**Electronic supplementary material:**

The online version of this article (doi:10.1186/s12951-014-0055-3) contains supplementary material, which is available to authorized users.

## Background

Cancer remains one of the leading causes of death. The majority of patients suffering from cancer undergo invasive treatments, such as surgery, radiation therapy and chemotherapy. Radiation therapy is based on the use of ionizing radiation to exterminate malignant cells via the production of free radicals that damage cellular DNA [1]. However, radio-toxicity to healthy tissue is a critical factor, because ionizing radiation does not well discriminate between malignant and normal cells [2,3]. Also chemotherapeutics do not exclusively act on malignant cells and exhibit side effects, mainly due to their poor specificity [4]. In this context, the hope for more selective alternatives has been revived by the advent of nanotechnology. In particular, gold nanoparticles (GNPs) have received considerable attention. In addition to their good biocompatibility, ease of preparation and stability, their optical features are ideal for applications in biomedical optics [5-10]. Their capacity to scatter and, more significantly, to absorb light results from localized plasmonic resonances [11-16].

Among the various shapes of GNPs, so-called gold nanorods (GNRs) exhibit two plasmonic bands, i.e. a weaker transversal band at ~ 520 nm, similar to that of gold nanospheres, and a more intense longitudinal band that moves from the visible to the near infrared (NIR) domains, say from 600 to 1100 nm, with increasing their aspect ratios [17-20]. Since tissue and skin components do not significantly absorb NIR light, GNRs are being proposed as contrast agents for many applications *in vivo* [21-24].

While polyethylene glycol (PEG) imparts very low cellular uptake [10], PEGylated GNRs tend to accumulate into tumours after intravenous injection much more than they do into normal tissue, because the vascular and lymphatic networks of neoplastic tissue are abnormal. This passive accumulation is known as the enhanced permeability and retention (EPR) effect. However, the fraction of GNRs that reaches tumours is quite low, say around 10%, while their entrapment in vital organs, such as the liver and the spleen, is substantial [25-29].

Various targeting units, such as antibodies [30-32], aptamers [33-35], peptides [36-38] and small molecules [39], have been anchored to the surface of GNRs, in an attempt to enhance their specificity for tumors. The interaction between these targeting units and their receptors on the membranes of malignant cells activates pathways of active uptake. The choice of molecular targets is critical. Popular receptors, such as folate [40-42] and growth factor receptors [43-45], are also found in most normal cells, and cause some undeliberate uptake from these non-targeted cells [46]. Nonspecific binding and specific binding to non-targeted cells are common nuisances. Some authors have proposed a dual-ligand approach to gain more specificity, especially when one of the molecular targets is rather unspecific [47-49]. In spite of all this effort, the classification of problems and bottlenecks in the systemic delivery of GNRs is hard, due to the extreme complexity of the biological interface.

In this paper, we propose an analytical approach to model *in vitro* some of the most critical issues that arise from the interaction between GNRs and the bloodstream. We focus on a single-ligand strategy, because the molecular target of our choice is Cancer Antigen 125 (CA125), which is very specific for ovarian cancers. CA125, also known as mucin 16, is the most reliable biomarker to confirm the diagnosis and the management of ovarian cancers, which is one of the most lethal gynaecological malignancies, and is a large molecular weight transmembrane glycoprotein.

We describe the preparation and the application of GNRs conjugated with anti-CA125 antibodies to detect cells overexpressing CA125 and mediate their selective photothermal ablation. The design of our probe starts from the PEGylation of GNRs with heterobifunctional PEG strands that confer biocompatibility, colloidal stability [10] and an easy dock for anti-CA125 antibodies. We place a special emphasis on the compatibility of these particles with intravenous injections, both in terms of their performances of molecular recognition and their interactions with erythrocytes and phagocytes. As for the formers, the threat of biological environments providing for competition and passivation is analyzed in solution by complementary tests with a quantitative profile. The qualitative translation of these findings into the cellular arena is confirmed by the specificity of anti-CA125 particles for HeLa cells, which are CA125- positive, even after incubation in biological fluids containing physiological levels of this antigen. Moreover, we address their haemolytic activity and their detection from macrophages, in an attempt to mimic the interactions occurring in the blood, liver, kidneys and spleen and exacerbating their blood clearance and organ sequestration. In Additional file 1, we provide evidence for the photothermal ablation of HeLa cells, thus confirming the efficacy and selectivity of the treatment.

Our results demonstrate that anti-CA125 GNRs are nontoxic, retain much of their ability of molecular recognition after incubation in biological fluids, do not compromise the erythrocytes and are not detected by the macrophages. For these reasons, bio-conjugated GNRs represent a promising platform for systemic delivery, in view of mini invasive imaging or therapeutic options based on concepts of photothermal or photoacoustic conversion.

## Results and discussion

### CTAB-capped GNRs

**Figure 1:**
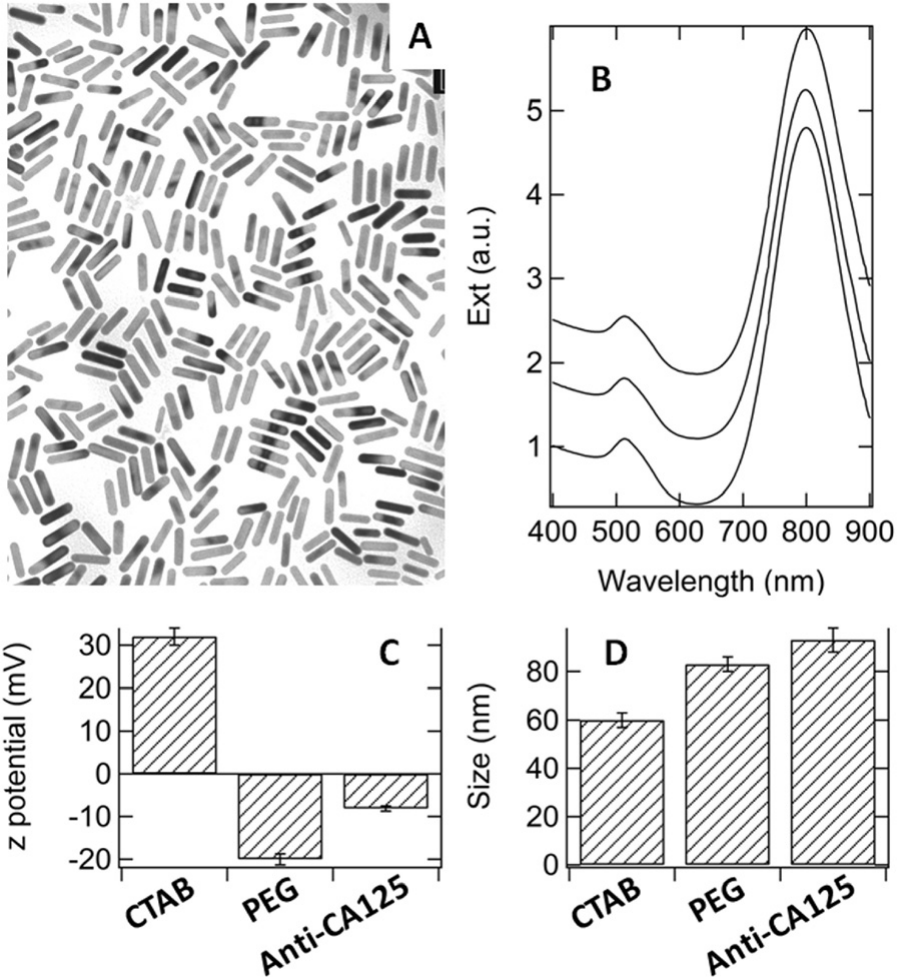
**Physical characterization. A)** Representative TEM image of CTAB-capped GNRs. **B)** Extinction spectra of CTAB-capped, PEGylated and anti-CA125 GNRs, respectively from bottom to top. **C)** Zeta potential and **D)** hydrodynamic diameter of CTAB-capped and surface-modified GNRs (in H_2_O). n = 8 for the DLS measurements.

As it is described in Methods, the preparation of our particles began with the synthesis of GNRs stabilized by hexadecyltrimethylammonium bromide (CTAB). TEM images of CTAB-capped GNRs revealed average lengths and widths of (43 ± 7) and (10 ± 3) nm, respectively (see Figure 1A). These particles exhibited a longitudinal plasmonic band around 800 nm (see Figure 1B).

### Anti-CA125-conjugated GNRs

Due to the toxicity of CTAB, the initial coating was substituted with a mixture of mono- and bi-functional PEG strands (methoxylated PEG, or mPEG, and carboxylated PEG, or cPEG), which are nontoxic polyether compounds in common use to improve the biocompatibility and systemic circulation of many particles [10,50,51]. The carboxy-terminals of GNRs were conjugated with antibodies anti-CA125, using the zero-length crosslinker 1-ethyl-3-(3-dimethylaminopropyl)carbodiimide (EDC) stabilized by N-hydroxysuccinimide (NHS) [52]. The reaction mechanism between cPEG and antibodies anti-CA125 involves the activation of the carboxy moieties of cPEG with EDC and NHS to form an unstable succinimide ester, which is prone to react with the amino moieties of antibodies to form a stable amide bond. As a confirmation of their successive modifications, zeta potential and hydrodynamic size measurements were performed by dynamic light scattering (DLS) on CTAB-capped GNRs, GNRs after immobilization of mPEG and cPEG mixtures and GNRs after conjugation with antibodies. The results are reported in Figure 1C and D. Their zeta potentials revealed the cationic [53], anionic and zwitteronic profiles of CTAB-capped, PEGylated and anti-CA125 GNRs, respectively. Likewise, their hydrodynamic radii underwent a progressive increase, which may be expected from the replacement of CTAB with PEG and then the addition of antibodies. On the other hand, the optical extinction of CTAB-capped, PEGylated and anti-CA125 GNRs showed negligible variations (Figure 1B), thus suggesting that these modifications preserved their plasmonic features.

### Cytotoxicity

In order to gain some preliminary insight into the biocompatibility of anti-CA125 GNRs, cell viability was evaluated *in vitro* in the presence of different doses of PEGylated or anti-CA125 GNRs on HeLa cells (see Figure A1 in Additional file 1). Anti-CA125 GNRs proved to be slightly more cytotoxic than PEGylated GNRs, probably because of their active targeting or the effect of antibodies per se. However, also anti-CA125 GNRs exerted little effect up to 100 μM Au.

### Specificity and environmental competition

A direct dot immunoassay was performed using GNRs with different modifications. The essential steps of this test involved the immobilization of CA125 on nitrocellulose membranes and its detection with anti-CA125 GNRs. This assay was developed to mimic an *in vitro* scenario, where CA125 is overexpressed on the surface of certain malignant cells and anti-CA125 GNRs are brought into contact with them. Staining occurred both with monoclonal antibody (mAb) anti-CA125 GNRs and polyclonal antibody (pAb) anti-CA125 GNRs, whereas GNRs conjugated with anti-rabbit immunoglobulins G (IgGs) did not adhere to the membranes, thus demonstrating the active role of the molecular recognition.

**Figure 2:**
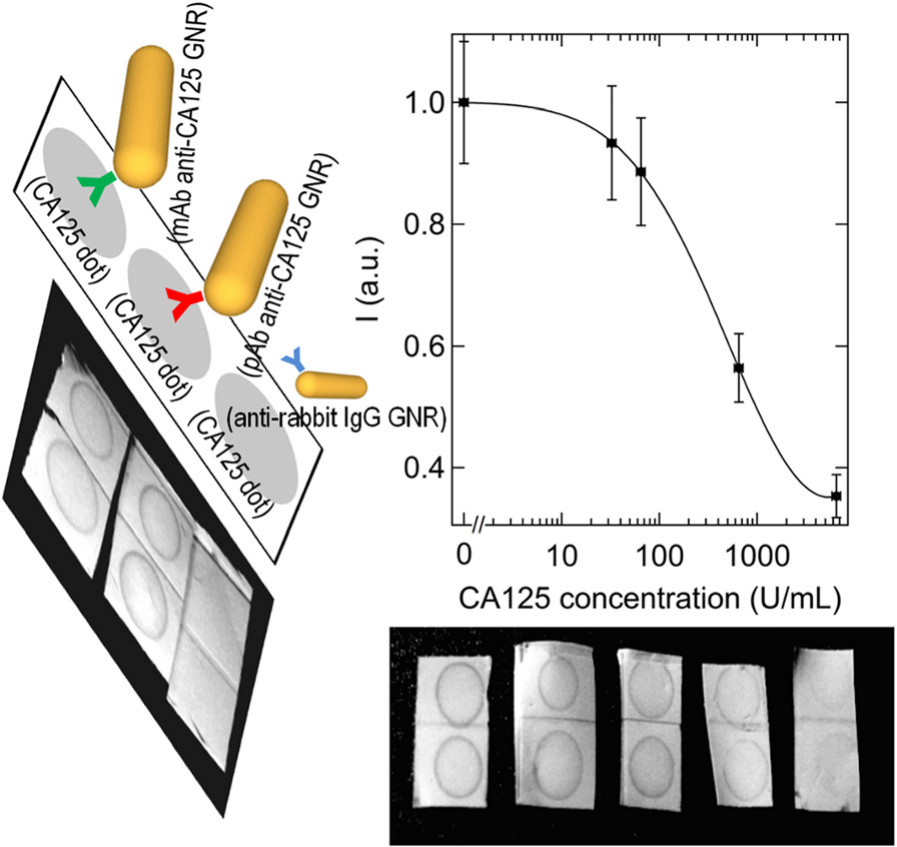
**Dot immunoassays.** On the left, schematic representation of a direct dot immunoassay performed using GNRs conjugated with different antibodies and a photograph of nitrocellulose membranes after incubation with various GNRs. Staining was only found in the presence of specific antibodies. On the right, calibration curve of a CA125 sensor based on a dot immunoassay with a competitive format and a photograph of a corresponding series of nitrocellulose membranes at the end of the test.

Furthermore, a dot immunoassay based on a competitive scheme was developed using mAb anti-CA125 GNRs. This assay was carried out to understand whether mAb anti-CA125 GNRs retain their ability to target their analyte even in the presence of free CA125. This circumstance mimics the *in vivo* conditions, where CA125 is present in the bloodstream, besides that on the surface of the malignant cells. In this case, the main steps of the assay involved the immobilization of CA125 on nitrocellulose membranes (at one given concentration), the incubation of a certain amount of anti-CA125 GNRs with standard solutions of CA125 (at different concentrations) and finally their interaction with the membranes. Staining of the membranes was found to decrease with an increase of free CA125 (Figure 2, on the right), consistent with the trend of a competitive assay. A dose–response curve for CA125 was retrieved by darkfield microscopy, from a quantitative measurement of the intensity of optical scattering from the particles (Figure 2, on the right). The average coefficient of variation among the various concentrations of free CA125 was found to be 10%. The signal began to decrease for CA125 concentrations higher than ~ 50 ppm and nicely followed a logistic behavior. The detection limit of this assay, i.e. the lowest concentration of free CA125 that was distinguishable from the absence of the analyte beyond statistical fluctuations, was ~ 90 ppm.

In practice, our immunoassay is unsuitable to discriminate healthy patients (CA125 in the range of 0 – 35 ppm) from those with pathological levels of CA125 (≥ 35 ppm). We note that the concentration of bio-conjugated GNRs that we used is not higher than the typical doses that are injected in the bloodstream for tests *in vivo* (say at least 10 mg Au per Kg animal [27,54-56] or 50 g blood, i.e. 1 mM Au in the blood). Therefore, our findings imply that the potential of anti-CA125 GNRs to target tumors is retained even in a regime of pathological conditions. In other words, our load of antibodies per particles is far from saturation even in a pathological environment. This result is not obvious, when it is considered that 400 μM Au amount to ~ 2 nM particles and 35 ppm CA125 corresponds to 40 – 150 nM CA125. At a glance, a lower limit for the number of recognition sites per particle must be ~ 20. In essence, anti-CA125 GNRs are good candidates to bind ovarian cancer cells *in vitro* and *in vivo.*

### Specificity and the matrix effect

**Figure 3:**
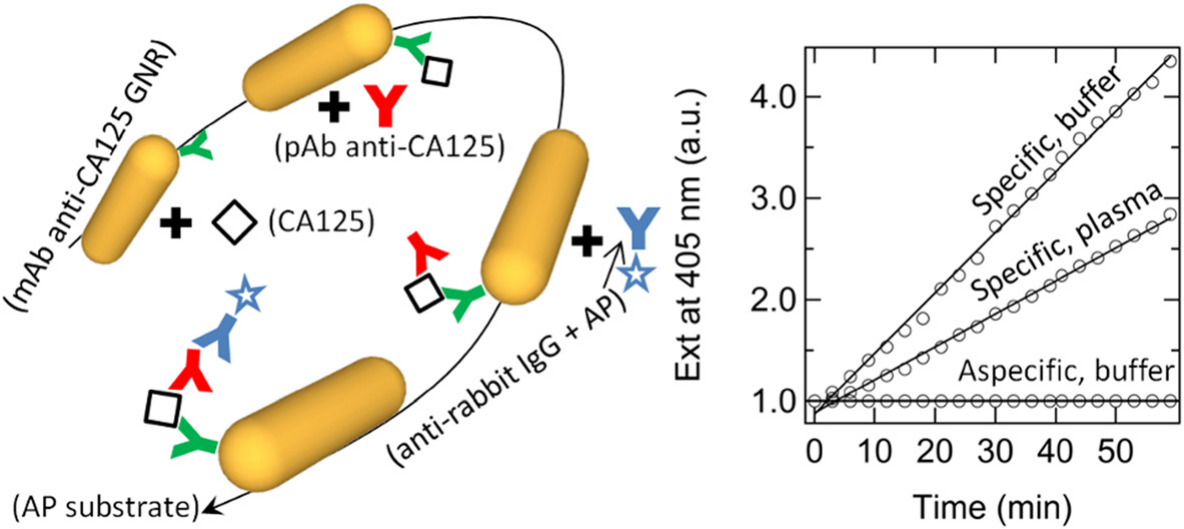
**Sandwich assays.** Left: schematic representation of the sandwich assay with enzymatic label performed on GNRs. Right: kinetics of the enzymatic reactions when GNRs modified with specific antibodies were incubated with a standard solution of CA125 (steepest line), human plasma (second steepest line) or when GNRs conjugated with aspecific antibodies (flattest line) were incubated with the same standard solution of CA125.

Another source of criticalities is the matrix effect. This effect was evaluated by a sandwich assay with an enzymatic label. A schematic representation of this assay is shown in the left panel of Figure 3. The rate of appearance of the enzymatic product (r) is proportional to the number of fundamental events of molecular recognition. Optical measurements in buffer solution and plasma were recorded over time (0 – 60 minutes), in order to quantify the enzymatic product. The kinetics of the enzymatic reactions are reported in the right panel of Figure 3. The slopes of the curves are similar in the cases when the analyte was dissolved in a standard solution (r ~ (6.0 ± 0.6)*10^−2^ s^−1^) or contained in a complex matrix such as plasma (r ~ (3.3 ± 0.3)*10^−2^ s^−1^). When CA125 was dissolved in the buffer solution, the kinetics was somewhat higher, which may be ascribed to various factors, including that the plasma could contain less than 30 ppm CA125 or also suppress any aspecific signal, due to the passivation given by the adsorption of plasmatic proteins. Anyway, the comparison between these two kinetics suggests that the matrix effect is not critical for these particles. The standard solution of CA125 was also incubated with GNRs modified with anti-bovine IgGs and the sandwich assay was run in order to gain an estimate of the extent of aspecific signal. In the right panel of Figure 3, this kinetics is compared with those obtained in the case of GNRs conjugated with specific antibodies. The incidence of aspecific signal (r ~ (3 ± 3)*10^−5^ s^−1^) proved to be negligible with respect to its specific counterpart.

### Specificity *in vitro* and the effect of biological fluids

The cellular uptake of GNRs was evaluated by darkfield microscopy, silver staining and spectrophotometry. The results of these three methods agreed on the effect of the surface modification on the uptake of GNRs by HeLa cells: PEGylated GNRs exhibited the lowest uptake, while anti-CA125 GNRs featured the highest uptake and specificity.

#### Darkfield microscopy

The plasmonic features of the GNRs are useful to reveal their cellular uptake by darkfield microscopy, which exploits the modulation of the optical scattering from a thin sample. This method has become a popular approach to identify GNRs in vitro [41,57], because of its noninvasive profile and suitability for dynamic inspections of living cells. After a preliminary characterization of the coefficients of optical scattering from the GNRs, the method described in refs. [57,58] was used. For each field of view, two darkfield images were acquired, spectrally filtered in and off the principal plasmonic resonance of the GNRs (780 nm high-pass and 510 nm bandpass filters). Then, after background subtraction, a pixel by pixel operation was performed to give a ratio image (*R* = I_780 nm_/I_510 nm_, where I is the intensity at the named wavelength). This ratio proved to be sensitive to the presence of GNRs.Three samples of HeLa cells were prepared, i.e. with overnight incubation of anti-CA125 GNRs or PEGylated GNRs and a blank sample without particles. In order to keep a focus on the cells, a mean value of *R* was calculated from individual cells or cell clusters ( ≈ 300) in each field of view and from various fields of view (≈ 10).

**Figure 4:**
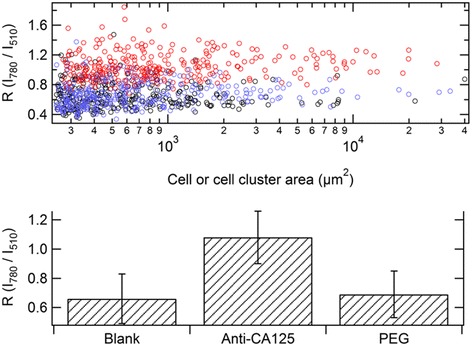
**Darkfield microscopy analysis.** Topmost panel: *R* value for each cell or cell cluster for each of the three samples as a function of the area of the cell or cell cluster (black, red and blue for the blank, anti-CA125 GNRs and PEGylated GNRs samples, respectively). Bottommost panel: the mean values of *R* for each of the three samples with their standard deviations.

Results are plotted in Figure 4. The mean value of *R* associated with the anti-CA125 GNRs sample (red circles, upper panel) was higher than those of the blank as well as the PEGylated GNRs samples (black and blue circles, respectively). A Student’s t-test was performed to qualify this observation, with the following results: p < 10^−5^ for both anti-CA125 GNRs – blank and anti-CA125 GNRs – PEGylated GNRs pairs; p = 0.53 in the case of the blank – PEGylated GNRs pair (~ 300 points for each sample). These figures are consistent with an accumulation of anti-CA125 GNRs and an absence of PEGylated GNRs.

#### Silver staining

Silver staining has become an option of choice for a qualitative assessment of the specificity of various gold nanoparticles in vitro, because of its convenience and sensitivity [36,43,59-65]. Here**,** metal particles nucleate the specific deposition of silver from an appropriate silver salt (silver acetate), in the presence of a suitable reducing agent (hydroquinone). Silver-coated particles then catalyze more silver deposition and so the silver grains grow in size and eventually become visible under a standard microscope. This principle was used to highlight the cellular uptake of anti-CA125 GNRs.

**Figure 5:**
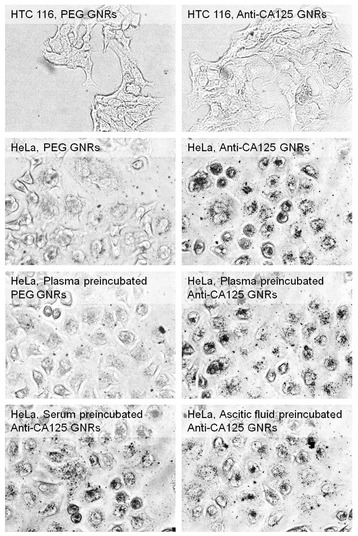
**Silver enhancement.** Evidence of targeting of CA125 via silver staining, using HCT 116 and HeLa cells as negative and positive models, respectively. Some samples of GNRs were preincubated with serum, plasma and ascitic fluid before their administration into the culture media.

HeLa and HCT 116 cells were treated with PEGylated GNRs or anti-CA125 GNRs. Figure 5 shows that only the specific GNRs/cell combination produced a significant precipitation of silver and thus a significant accumulation of particles. For HCT 116 cells, no deposition of silver was observed for either kind of GNRs. Instead, for HeLa cells, a high precipitation of silver was only observed in the case of anti-CA125 GNRs and this was well confined within the cells. The comparison between anti-CA125 GNRs and PEGylated GNRs was corroborated with a quantitative spectrophotometric analysis [60], which gave a ratio between the extent of specific to aspecific uptake from HeLa cells in the order of 6 ± 3 (see Figure A2 in Additional file 1).

Finally, we verified the translation of our findings on the interplay of environmental competition and matrix effect *in vitro*. Particles were incubated with critical examples of biological fluids (serum, plasma or ascitic fluid from mice bearing ovarian cancers, at a representative rate of 400 μM Au) for one hour and then left to interact with HeLa cells. Even after this treatment, only anti-CA125 GNRs were found to retain a significant uptake from HeLa cells, although some attenuation of the pristine contrast with their PEGylated counterpart is visible. This result confirms that the interaction with free analyte in biological tissue does not saturate the antibodies on the surface of the particles, consistent with the outcome from our dot immunoassays. In addition, the specificity of anti-CA125 GNRs without and with preincubation in biological fluids was found to correlate well with the efficiency of a hyperthermic effect based on an optical treatment, as it is discussed in Additional file 1 (see Figure A3).

### Haemolysis and detection from macrophages

**Figure 6:**
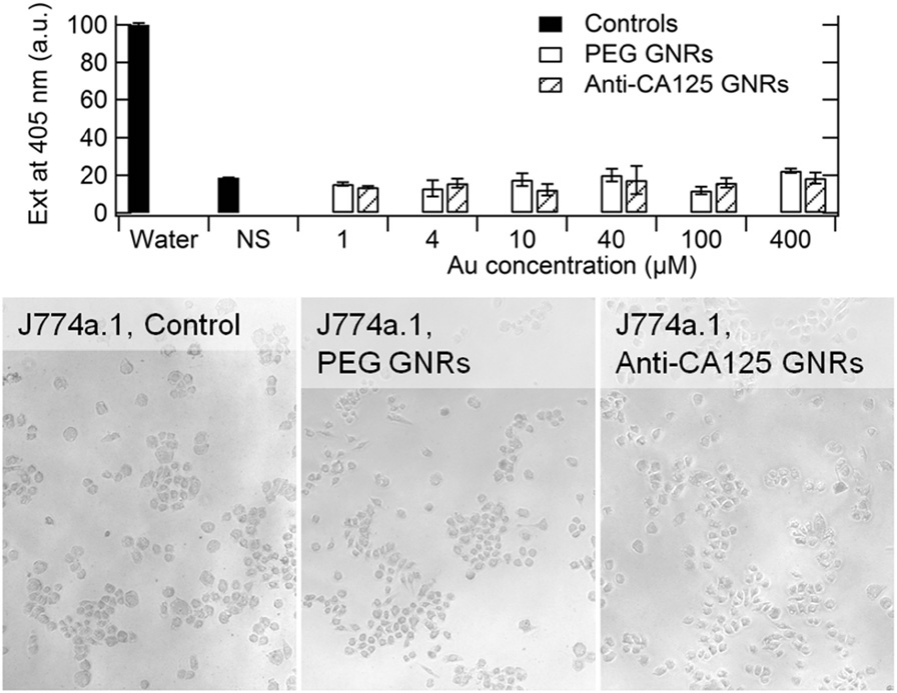
**Interactions with erythrocytes and macrophages.** Upper part: the degree of haemolysis produced by anti-CA125 GNRs at various concentrations is reported and compared with that produced by PEGylated GNRs. Data are referred to positive controls that were obtained by dosing ultrapure water to induce a complete haemolysis. Lower part: the absence of silver deposition shows that particles are not internalized by macrophages.

In order to complement our analysis on the suitability of anti-CA125 GNRs for an intravenous administration, we assessed their haemolytic activity. Erythrocytes were incubated with a positive control (ultrapure water), a negative control (normal saline) or various concentrations of anti-CA125 GNRs or PEGylated GNRs. According to the topmost panel of Figure 6, anti-CA125 GNRs exhibited the same behavior as PEGylated GNRs and both types of particles displayed no haemolytic activity, even at their highest concentrations. Data are referred to positive controls obtained by dosing ultrapure water to induce a complete haemolysis.

Moreover, we implemented a phagocytosis assay, because this parameter may impair the ability of anti-CA125 GNRs to remain in the bloodstream by their sequestration from phagocytes. The amount of gold internalized by macrophages exposed to anti-CA125 GNRs or PEGylated GNRs at a concentration of 100 μM Au was probed by silver staining after an overnight incubation. Here, 12 – 18 hours are regarded as an upper limit for the persistence of bio-conjugated GNRs in the bloodstream [44]. As shown in the bottommost panel of Figure 6, on visual inspection, no precipitation of silver was found for the PEGylated GNRs sample, as it is expected from the literature [10,28], but also for the anti-CA125 GNRs sample. Therefore, the addition of anti-CA125 antibodies did not jeopardize the stealth profile imparted by the PEG portion.

## Conclusions

In conclusion, we have presented a model of anti-CA125-conjugated gold nanorods that are intended to target ovarian cancer cells after intravenous injection. We have combined a battery of tests to understand their specificity for surfaces and cells overexpressing CA125, as well as possible issues that may arise from their interface with a bloodstream, both in terms of their performances of molecular recognition and their compatibility with erythrocytes and phagocytes. We have found that both the competition from free CA125 in biological fluids and the matrix effect affected the ability of our particles to detect their biochemical target to a moderate extent. This modulation was analytically quantified in solution and qualitatively confirmed in cells. In addition, our particles were not harmful to erythrocytes and did not suffer from massive phagocytosis and sequestration from macrophages, such as those residing in the liver, kidneys and spleen.

With these premises, our particles are a meaningful candidate for future investigations with animal tissue *ex vivo* and *in vivo*. While this analysis does not intend to replace animal testing, we are confident that our approach may inspire additional efforts to enhance the preliminary screening of functional particles that are directed to systemic administration. Our future work will aim to refine the dependability of our models and the quantitative profile of our predictions on the interactions between particles and their biological targets in the presence of a circulatory system.

## Methods

### Materials

HAuCl_4_ (hydrogen tetrachloroaurate (III) hydrate), CTAB (hexadecyltrimethylammonium bromide), NaBH_4_ (sodium borohydride), ascorbic acid, silver nitrate, NHS (N-hydroxysuccinimide), EDC (1-ethyl-3-(3-dimethylaminopropyl)carbodiimide), polysorbate 20, paraformaldehyde, silver acetate, hydroquinone, trypsin, trypan blue, anti-rabbit IgG labelled with alkaline phosphatase, 4-nitrophenyl phosphate bis(cyclohexylammonium) salt, milk powder, human serum, human plasma and MTT (3-(4,5-dimethylthiazol-2-yl)-2,5-diphenyltetrazolium bromide) assay kit as well as all chemicals for the various buffer solutions were purchased from Sigma Aldrich. Monoclonal mouse antibody anti-CA125 (mAb anti-CA125), polyclonal rabbit antibody anti-CA125 (pAb anti-CA125) and CA125 partial recombinant protein were purchased from Novus Biologicals. Alpha-methoxy-omega-mercapto-poly(ethylene glycol) (mPEG-SH) and alpha-carboxy-omega-mercapto-poly(ethylene glycol) (cPEG-SH), M_w_ ≈ 5000 gmol^−1^, were provided by Iris Biotech. All cell culture media, fetal calf serum and antibiotics (penicillin and streptomycin) were purchased from Gibco. All chemicals were of analytical grade. Nitrocellulose membranes with pore size of 0.45 μm were purchased from Whatman.

### Cell lines and culture conditions

Human colon colorectal carcinoma cells (HCT 116) (negative line, i.e. not expressing CA125), human cervix carcinoma cells (HeLa) (positive line, i.e. overexpressing CA125) and murine macrophages were used. All cell lines were maintained in Dulbecco Modified Eagle Medium (DMEM) supplemented with fetal bovine serum, 100 units/mL penicillin, and 100 μg/mL streptomycin and kept under standard culture conditions (37°C, 5% CO_2_, 95% air and 100% relative humidity).

### Instrumentation

Optical spectra of aqueous suspensions of GNRs were measured by a UV-NIR spectrophotometer (V-560, Jasco, Japan). Their zeta potential and hydrodynamic size were characterized by Dynamic Light Scattering (DLS, Zetasizer Nano-ZS90, Malvern Instruments, UK). Imaging was performed by Transmission Electron Microscopy (TEM, CM12, Philips, the Netherlands) or optical microscopy operated in darkfield or standard conditions. For TEM, GNRs were left to dry on carbon-coated films and imaged at 100 kV. The darkfield microscope consisted of a Nikon (Japan) Eclipse TE-2000 platform equipped with a Nikon darkfield condenser (immersion oil, min NA = 1.2, max NA = 1.4), a Nikon 10 × objective (NA = 0.3), a set of optical filters (510 nm, 40 nm FWHM passband filter XF3043, Omega Optical, USA and 780 nm highpass filter OG780, Schott AG, Germany) and a CCD camera (Coolsnap-HQ2, Roper Scientific, USA). Cells were also observed with a Leica (Germany) DMI3000B inverted microscope. The optical excitation described in Additional file 1 was performed with a low power 810 nm diode laser (Weld 800, El.En., Italy).

### Synthesis of GNRs

CTAB-capped GNRs were synthesized by the autocatalytic reduction of HAuCl_4_ with ascorbic acid, according to the method proposed by Nikoobakht et al. [66], with the variant and overgrowth by Ratto et al. [18].

### PEGylation of GNRs

After purification by two cycles of centrifugation and decantation with a dead volume ratio of ~ 1/200, GNRs were transferred at a concentration of 1.6 mM Au into a 100 mM acetate buffer at pH 5 containing 500 μM cetrimonium bromide and 5 μM cPEG-SH. This suspension was left to react at 37°C for 30 minutes and then 50 μM mPEG-SH was added and kept at rest for another 90 minutes. After purification, GNRs were transferred at a concentration of 1.6 mM Au into a 10 mM MES buffer at pH 6 containing 120 mM NaCl and 0.005% (v/v) polysorbate 20. The M_w_ of PEG of ≈ 5000 gmol^−1^ was chosen to provide for high colloidal stability, low aspecific interactions with cells [10] and so the perspective to take full advantage of the EPR effect [67].

### Preparation of anti-CA125-conjugated GNRs

An equal volume of a solution containing 12 mM NHS and 48 mM EDC was added to a suspension of GNRs at a concentration of 1.6 mM Au in 10 mM MES buffer at pH 6. After 15 minutes of activation, this suspension was incubated with a double volume of 20 ppm Ab anti-CA125 in MES buffer at pH 6 containing 120 mM NaCl and 0.005% (v/v) polysorbate 20. After one hour, 10 mM 2-methoxyethylamine was dosed for 30 minutes, in order to block any unreacted succinimide ester. After purification by two cycles of centrifugation and decantation with a dead volume ratio of ~ 1/200, GNRs were transferred at a concentration of 4.0 mM Au into sterile PBS.

### Dot immunoassay

Dot immunoassays were performed using 0.45 μm pore size nitrocellulose membranes. In a typical protocol of a direct assay, 1 μL of 1000 ppm CA125 partial recombinant protein in carbonate buffer at pH 9.6 was spotted onto a membrane. Then, the spot was left to dry in an oven at 37°C for 20 minutes. Nonspecific binding was inhibited by incubation of the membrane for 40 minutes at room temperature in a blocking PBS buffer containing 3% (w/v) milk powder. Then, the membrane was incubated for one hour at room temperature and under gentle stirring with 500 μL of a suspension of 400 μM Au mAb anti-CA125 GNRs, pAb anti-CA125 GNRs or anti-rabbit IgG GNRs. Finally, the membrane was washed twice with a PBS buffer containing 0.1% (v/v) polysorbate 20 and left to dry at room temperature.

For a competitive assay, nitrocellulose membranes were spotted with CA125 and blocked, as in the case of a direct assay. Meanwhile, 400 μM Au mAb anti-CA125 GNRs were incubated for one hour with standard solutions containing variable concentrations of CA125, in the range 0 – 5000 ppm. After purification, GNRs were resuspended at a concentration of 400 μM Au in 1 mL of PBS buffer and incubated with the membranes for one hour at room temperature and under gentle stirring. Finally, the membranes were rinsed with abundant PBS buffer containing 0.1% (v/v) polysorbate 20 and left to dry at room temperature. The readout was devised as a quantitative light scattering measurement by darkfield microscopy, which reflects the amount of GNRs bound to the membranes.

### Sandwich assay

50 μL of mAb anti-CA125 GNRs at a concentration of 4.0 mM Au in PBS buffer were added to 450 μL of PBS buffer supplemented with 30 ppm CA125 or 450 μL of human serum or plasma containing their physiological level of CA125. After one hour of incubation at 37°C, GNRs were purified and resuspended at a concentration of 4.0 mM Au in 50 μL of PBS buffer and incubated for one hour at 37°C with 450 μL of PBS buffer containing 20 ppm pAb anti-CA125 that had formerly been left to react for 30 minutes with anti-rabbit IgG labelled with alkaline phosphatase (1/20000 of its stock solution). After purification, 50 μL of GNRs at a concentration of 4.0 mM Au were transferred into 450 μL of 2.4 mM 4-nitrophenyl phosphate bis(cyclohexylammonium) salt in DEA buffer. The alkaline phosphatase enzyme catalyzed the formation of a soluble end product that was bright yellow. This reaction was monitored with a spectrophotometer at 405 nm.

### Measurement of cellular uptake

Cellular uptake of the mAb anti-CA125 GNRs was evaluated using three different techniques, i.e. darkfield microscopy, silver staining and spectrophotometry. HeLa and HCT 116 cells were seeded and allowed to grow for 24 hours in 24-well culture plates or on glass coverslips. Cells were then treated overnight with mPEG GNRs (non-targeted particles) and mAb anti-CA125 GNRs (targeted particles) at a concentration of 100 μM Au in culture medium. Untreated cells served as a background control. Alternatively, some aliquots of mPEG GNRs and mAb anti-CA125 GNRs at a concentration of 400 μM Au were exposed to human serum, plasma or murine ascitic fluid for one hour, centrifuged and decanted, prior to their incubation with the cells at a concentration of 100 μM Au in culture medium. The day after, cells were washed with abundant PBS in order to remove all unbound GNRs.

For darkfield microscopy and silver staining, cells were fixed in a solution of 3.6% paraformaldehyde in PBS buffer for 5 minutes and washed with PBS buffer to remove the excess of reagents. For a qualitative inspection by silver staining, samples were incubated for 5 minutes with 23 mM hydroquinone in citrate buffer at pH 3.8 and then for 4 – 18 minutes with the same solution supplemented with 6 mM silver acetate. All solutions were as fresh as possible. Samples were observed with a standard microscope. For a quantitative optical analysis, cells were counted, centrifuged and suspended in 120 μL of DI water, before inspection with a spectrophotometer (see Figure A2 in Additional file 1).

### Measurement of interactions with erythrocytes and macrophages

For the evaluation of haemolysis, informed signed consent was obtained and human whole blood was collected from healthy volunteers. Test tubes containing 1.8 mg/mL EDTA were used to collect the whole blood. Samples were centrifuged at 3000 rpm for 20 minutes and the buffy coat was collected and washed with normal saline. 100 μl of samples diluted with normal saline to a 50% hematocrit were added to 3 mL of normal saline (as a negative control), ultrapure water (as a positive control) and suspensions of anti-CA125 GNRs in PBS buffer at different concentrations. All samples were incubated at 37°C for one hour and haemolysis was stopped by the addition of 50 μl of 2.5% glutaraldehyde, prior to centrifugation at 3000 rpm for 15 minutes. Supernatants were collected in 96-well microplates. Their absorbance was measured at 405 nm by an automated plate reader.

The uptake of anti-CA125 GNRs from macrophages was visualized by silver staining.

## Additional file

Additional file 1:
**Cytotoxicity, quantitative measurement of cellular uptake, optical hyperthermia in vitro.** This file contains additional information on the biological profiles and functional properties of anti-CA125 GNRs [68-71].
