# Supplementary material for: In vitro assessment of antibody-conjugated gold nanorods for systemic injections
Source: J Nanobiotechnology. 2014 Dec 5;12:55. doi: 10.1186/s12951-014-0055-3 (PMC4266900; doi:10.1186/s12951-014-0055-3)
Supplement: Additional file 1: — Cytotoxicity, quantitative measurement of cellular uptake, optical hyperthermia in vitro. This file contains additional information on the biological profiles and functional properties of anti-CA125 GNRs [68-71]. [file 12951_2014_55_MOESM1_ESM.doc]

**Additional file 1**

**In vitro assessment of antibody-conjugated gold nanorods for systemic injections**

**Sonia Centi^†^, Francesca Tatini^‡^, Fulvio Ratto^‡,*^, Alessio Gnerucci^†^, Raffaella Mercatelli^§^, Giovanni Romano^†^, Ida Landini^ǁ^, Stefania Nobili^ǁ^, Andrea Ravagli^§^, Giovanna Marrazza^§^, Enrico Mini^ǂ^, Franco Fusi^†^ and Roberto Pini^‡^**

**^†^** Dipartimento di Scienze Biomediche Sperimentali e Cliniche 'Mario Serio', Università degli Studi di Firenze, Viale Pieraccini 6, 50139 Firenze (Italy)

**^‡^** Istituto di Fisica Applicata 'Nello Carrara', Consiglio Nazionale delle Ricerche, Via Madonna del Piano 10, 50019 Sesto Fiorentino (Italy), Email: f.ratto@ifac.cnr.it, Phone: +39 055 5225307, Fax: +39 055 5226201

**^§^** Dipartimento di Chimica 'Ugo Shiff', Università degli Studi di Firenze, Via della Lastruccia 3, 50019 Sesto Fiorentino (Italy)

**^ǁ^** Dipartimento di Scienze della Salute, Università degli Studi di Firenze, Viale Pieraccini 6, 50139 Firenze (Italy)

**^ǂ^** Dipartimento di Medicina Sperimentale e Clinica, Università degli Studi di Firenze, Largo Brambilla 3, 50134 Firenze (Italy)

**Cytotoxicity.** Exponentially growing cells were inoculated into 96-well microplates and maintained under standard culture conditions for 24 h. Thereafter, the medium was removed and replaced with fresh medium containing different concentrations of anti-CA125 gold nanorods (GNRs) and GNRs with a mixture of mPEG and cPEG, activated and then blocked with methoxyethylamine (PEGylated GNRs). After 24 hours, an MTT reduction assay was performed. Cells were incubated with a 0.5 mg/mL MTT solution at 37°C for 4 hours and then with a cell lysis buffer (20% SDS, 50% N,N-dimethylformamide at pH 4.7) for 3 hours. The optical absorbance of blue formazan was determined at 570 nm by an automated plate reader.

Figure A1 displays the results of the cytotoxicity measurement, 24 hours after the addition of anti-CA125 GNRs and PEGylated GNRs at various concentrations. The cell viability was expressed as the percentage of MTT reduction in treated cells with respect to control cells. Data are reported as mean ± standard deviation of three independent experiments. Once the atoxicity of the antibodies per se was checked, we found that PEGylated GNRs did not affect HeLa cells at concentrations ranging from 4 to 100 μM Au and, only when applied at higher rates (400 μM Au), triggered a moderate inhibition of cell viability. Instead, mAb anti-CA125 GNRs began to exhibit a slight cytotoxic effect from above 10 μM Au, probably due to their targeting effect.

**
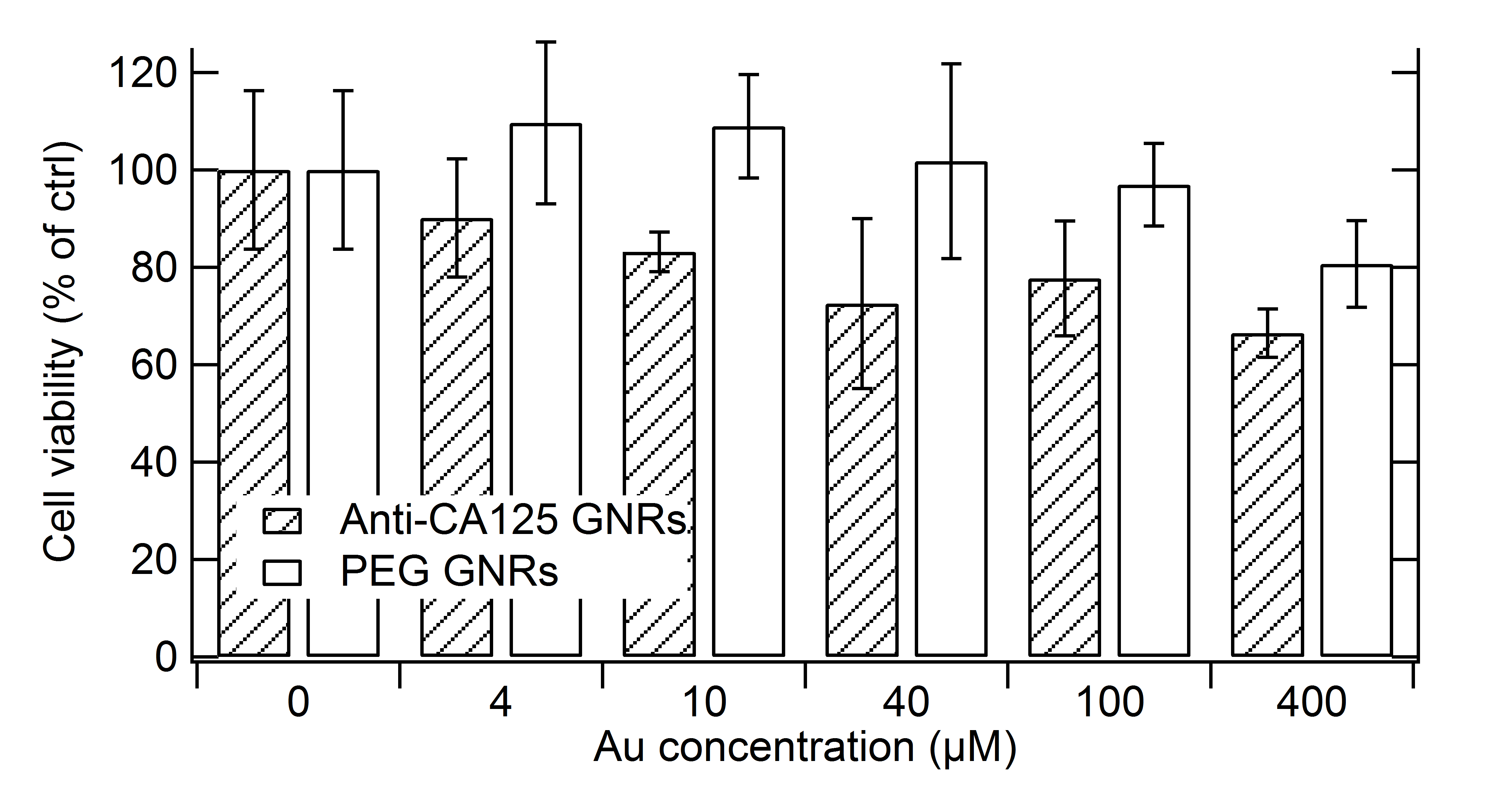
**

Figure A1: MTT reduction test for cell viability on HeLa cells after 24 h exposure to PEGylated and anti-CA125 GNRs. Cell viability is expressed as percent of MTT reduction in treated cells with respect to control cells. Reported data represents the mean ± standard deviation of three independent experiments.

**Quantitative measurement of cellular uptake.** The accumulation of anti-CA125 GNRs in HeLa cells was quantified by the analysis of optical measurements that exploited their plasmonic features [60]. For the optical measurements, cells were plated into 60 mm diameter petri dishes and allowed to recover overnight. Then, mAb anti-CA125 GNRs or PEGylated GNRs at a concentration of 100 µM Au were added and cells were kept at rest for another 24 hours. Thereafter, cells were fixed, washed several times, reconstituted in 120 µl of DI water in a quartz microcuvette and analyzed by a Jasco V-560 spectrophotometer. Their spectra of optical extinction were modeled as the sum of two contributions, i.e. an empirical background from an unknown number of cells, which was calibrated in a preliminary experiment, plus a numerical approximation of the plasmonic band from an unknown quantity of gold nanorods. The latter was devised as a convolution integral between Gans lineshape [68-70] using the dielectric function of gold by Etchegoin et al. [71] and an unknown distribution of particle shapes. Details of this method are provided elsewhere [60,69,70]. Here, the volume fraction of gold returned by the computation was corrected by an empirical factor that compensates for the various approximations of the numerical model, which was retrieved from a preliminary analysis of a standard suspension. With this factor, the output of this model conveys a quantitative rate of gold taken up per cell.


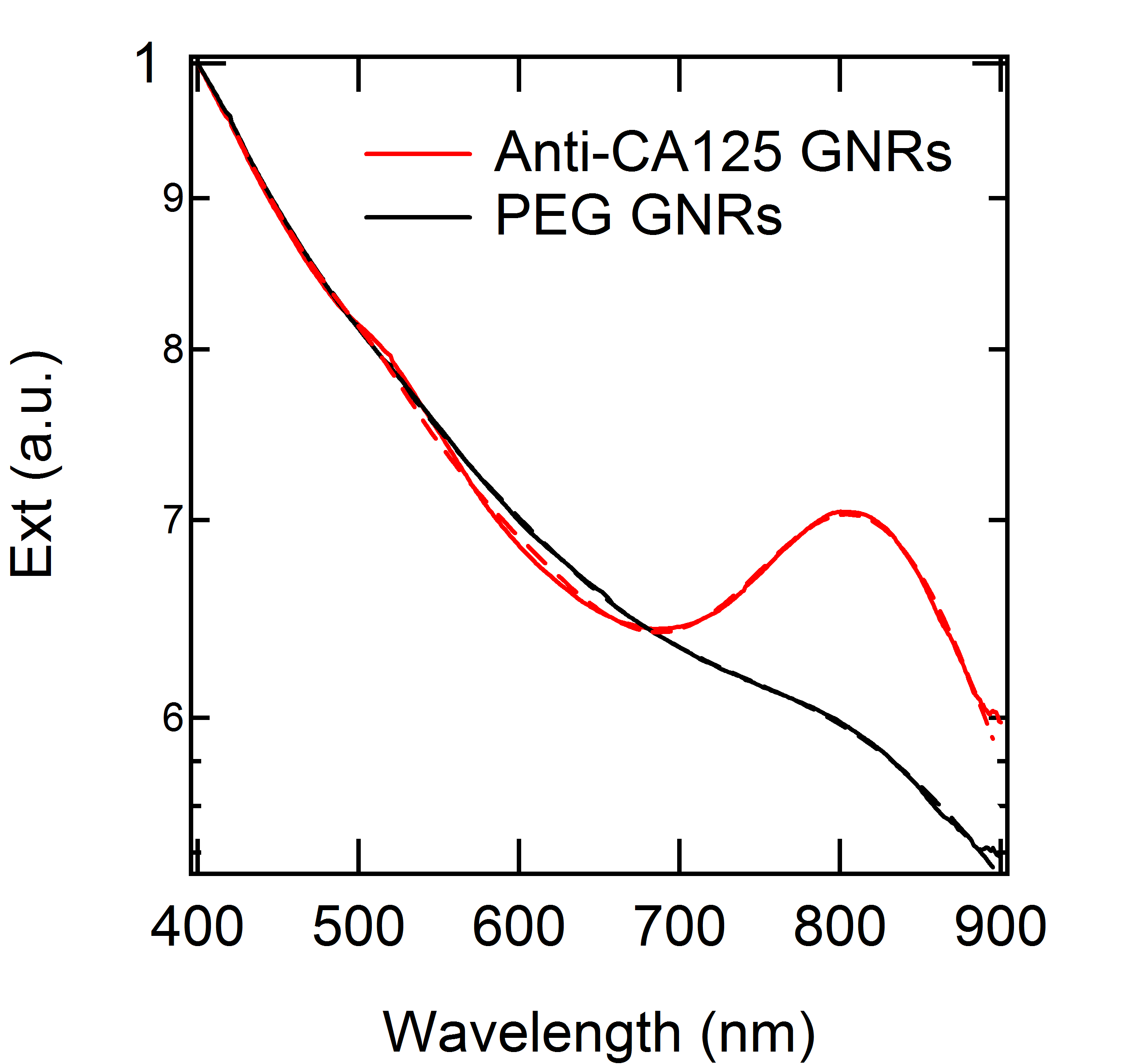


Figure A2. Optical analysis of HeLa cells treated with mAb anti-CA125 GNRs (red lines) or PEGylated GNRs (black lines). The experimental data (continuous lines) are overlapped with their numerical model (broken lines).

According to Fig. A2, we found that the cells treated with anti-CA125 GNRs exhibited a clear plasmonic band around 800 nm, which was ascribed to the presence of (12 ± 2) pg Au per cell. Conversely, the cells incubated with mPEG GNRs only displayed a weak modulation in the NIR window, which was quantified as (2 ± 1) pg Au per cell.

**Optical hyperthermia *in vitro*.** The specificity of anti-CA125 GNRs for HeLa cells is intended to convey their sensitization to an optical treatment in resonance with the plasmonic oscillations of the particles. Cells were allowed to grow in 33 mm diameter petri dishes until a confluence of 80 – 90%. Optical treatments were performed with a bench setup consisting of an AlGaAs diode laser coupled with a 600-μm-core optical fiber and a heating stage to keep the base temperature at 37 °C. 4 hours after the optical excitation, cell death was checked by staining with trypan blue. Damaged cells are not able to excrete the dye and so display a blue color under a standard microscope.

Figure A3 suggests the therapeutic potential of the anti-CA125 GNRs. The exposure to NIR light of malignant cells incubated overnight with anti-CA125 GNRs or PEGylated GNRs was performed for 10 minutes with a few power densities in the range 50 – 300 W/cm^2^. Cell death started from ~ 100 W/cm^2^ in the case of anti-CA125 GNRs, while 300 W/cm^2^ were barely enough to affect the cells treated with PEGylated GNRs. Also in the case of GNRs preincubated with biological samples (serum or ascitic fluid), only anti-CA125 GNRs mediated a large extent of cell death, consistent with our results on their cellular uptake. Figure A3 shows representative images of cells in the irradiated areas.

**
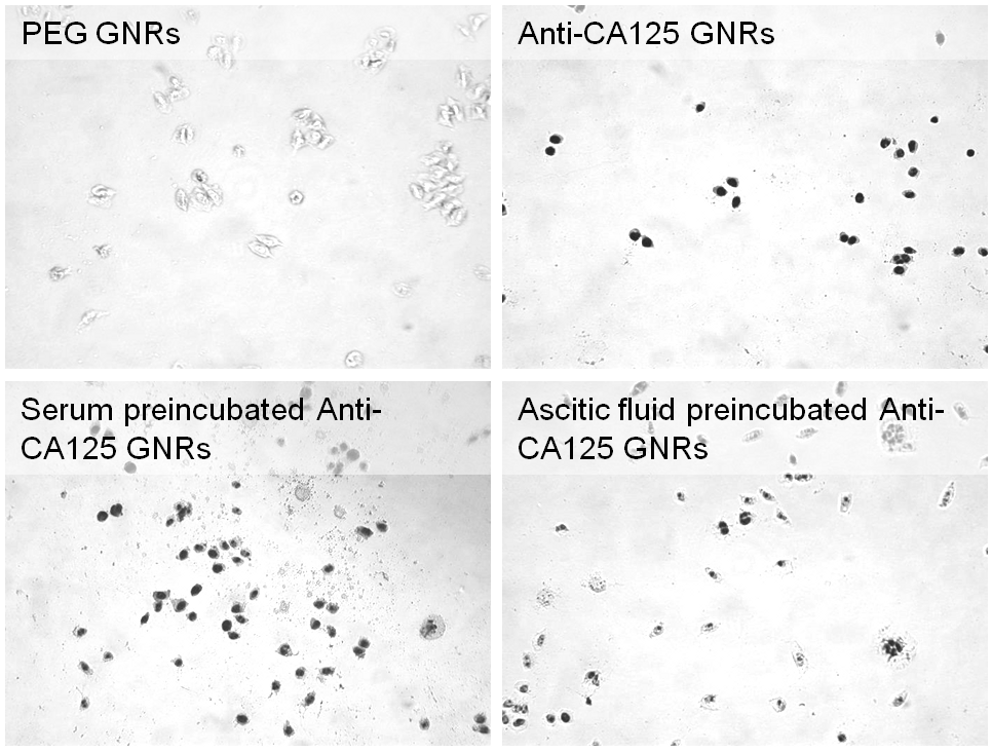
**

| Figure A3: Trypan blue staining of HeLa cells after irradiation with NIR light for 10 minutes at a power density of 100 W/cm^2^. Some samples of GNRs, before incubation with the cells, were preincubated with serum and ascitic fluid. |
| --- |
